# Profiling of a novel circadian clock-related prognostic signature and its role in immune function and response to molecular targeted therapy in pancreatic cancer

**DOI:** 10.18632/aging.204462

**Published:** 2023-01-09

**Authors:** Yu Jin, Shuang Gong, Guochen Shang, Lilin Hu, Gangping Li

**Affiliations:** 1 Division of Gastroenterology, Union Hospital, Tongji Medical College, Huazhong University of Science and Technology, Wuhan 430022, China; 2 First School of Clinic Medicine, Tongji Medical College, Huazhong University of Science and Technology, Wuhan 430022, China

**Keywords:** circadian clock, neutrophil, pancreatic cancer, prognosis, targeted therapy

## Abstract

Background: Pancreatic ductal adenocarcinoma (PADA) represents a devastating type of pancreatic cancer with high mortality. Defining a prognostic gene signature that can stratify patients with different risk will benefit cancer treatment strategies.

Methods: Gene expression profiles of PADA patients were acquired from the Cancer Genome Atlas and Gene Expression Omnibus, including GSE62452 and GSE28735. Differential expression analysis was carried out using the package edgeR in R. Intro-tumor immune infiltrates were quantified by six different computational algorithms XCELL, TIMER, QUANTISEQ, MCPCOUNTER, EPIC, and CIBERSORT. Biological processes were investigated based on R package “clusterProfiler”.

Results: 13 genes (*ARNTL2, BHLHE40, FBXL17, FBXL8, PPP1CB, RBM4B, ADRB1, CCAR2, CDK1, CSNK1D, KLF10, PSPC1, SIAH2*) were eligible for the development of a prognostic gene signature. Performance of the prognostic gene signature was assessed in the discovery set (n = 210), validation set (n = 52), and two external data set (GSE62452, n = 65, and GSE28735, n = 84). Area under the curve (AUC) for predicting 3-year overall survival was 0.727, 0.732, 0.700, and 0.658 in the training set, the validation set, and the two test sets, respectively. KM curve revealed that the low-risk group had an improved prognosis than the high-risk group in all four datasets. PCA analysis demonstrated that the low-risk group was apparently separated from the high-risk group. CD8 T cell and B cell were significantly reduced in the high-risk group than in the low-risk group, while neutrophils were significantly augmented in the high-risk group than in the low-risk group. BMS-536924, Foretinib, Linsitinib, and Sabutoclax were more sensitive in the low-risk group, whereas Erlotinib was more effective in the high-risk group.

Conclusions: We successfully established and verified a novel circadian clock-related gene signature, which could stratify patients with different risk and be reflective of the therapeutic effect of molecular targeted therapy. Our findings could incorporate the pharmacological modulation of circadian clock into future therapeutic strategies.

## INTRODUCTION

Pancreatic ductal adenocarcinoma (PADA) accounts for approximately ninety percent of pancreatic cancers, and represents a lethal cancer, with an estimated 232000 newly diagnosed cases, 22700 cancer-associated mortality annually, and a less than 5% five-year survival rate [1, 2]. Current standard treatment for PADA is a combination therapy using chemotherapy, molecular targeted therapy, immunotherapy and surgical operation. Unfortunately, despite advancement in therapeutic strategies, a median survival time for PADA patients is only 23 months. On the other hand, we should pay attention to this phenomenon that approximate 27% of patients with resected PDAD can survive for five years. One underlying rationale for this phenomenon is heterogeneity of tumor phenotypes of pancreatic cancer itself. Another potential explanation might be that some patients' response to some treatment regiment, while the others fail to response to the therapy strategies. Therefore, it seems essential to distinguish between patients who would most benefit from the current therapy and those who would be not fit for the treatment [3, 4]. To this end, the present study aimed to define an indicator of distinct survival outcomes.

The circadian clock endows humans the ability to perceive temporal changes of the external environment through a series of physiological activities [5]. The circadian rhythm is a sophisticated biological process, and supervises a series of cyclic biological functions via regulating a diversity of molecular activity and signal transduction processes [6]. In the case that circadian clock is disrupted, the hazard of conditions such as metabolic disorders, cardiovascular disease and cancer will increase [1]. Literatures have revealed a link between circadian rhythm disturbance and human cancer [7]. Although the negative effect of circadian clock disruption has gradually begun to be known [8–10], it remains underappreciated to exploit it for making more effective chronotherapy strategies. Besides, the role of circadian clock in PADA is elusive; therefore, we sought to interrogate the specific association between circadian clock and the pathogenesis of PADA.

In addition, PADA is an exceptionally heterogeneous carcinoma [11], with different phenotype in distinct cases [4, 12]. In despite of immune therapy has been applied for the treatment for cancers [13, 14], the detailed rationale of the role of immunotherapy in PADA is largely to be explored. Emerging data revealed that it is the crosstalk between cancer cells and intro-tumor immune infiltrates that play a pivotal role in the tumorigenesis and progression of cancer [15, 16]. Cytotoxic immune cells such as CD8+ T cell, as well as immune transduction processes are impaired in the intra-tumor microenvironment [7]. Nevertheless, the precise and exact processes of them are still required to be interrogated.

Facing the challenges in the process of treating PADA that how we could precisely distinguish between the low- and high- risk cohorts and how the immune infiltrates crosstalk to cancer cells, we aim to develop an indicator of distinct prognostic outcomes, and investigate its association with immune function and therapeutic efficacy, hoping to incorporate the pharmacological modulation of circadian clock into future therapeutic strategies.

## MATERIALS AND METHODS

### Acquisition of data

Gene expression information of PADA patients was acquired from the Cancer Genome Atlas (TCGA) database and Gene Expression Omnibus (GEO), including GSE62452 [17] and GSE28735 [18]. TCGA contains 172 PADA patients with a follow-up time of more than one month, among including of 171 primary tumors and one metastatic tumor. These samples were divided into the discovery set (n = 120) and validation set (n = 52). GSE62452 contains 69 PADA and adjacent non-tumor samples, among which 65 PADA samples had a follow-up time of more than one month. These samples were snap-frozen and sequenced using Affymetrix Human Gene 1.0 ST Array (GPL6244). GSE28735 contains 45 PAD patients, among which 42 PADA patients had a follow-up time of more than one month. These samples were also snap-frozen and sequenced using Affymetrix Human Gene 1.0 ST Array (GPL6244). Gene expression information was got through Cancer Cell Line Encyclopedia (CCLE) database. IC50 values of pancreatic cancer cell lines was got from Genomics of Drug Sensitivity in Cancer (GDSC). In the present study, gene expression profiling from TCGA was subjected to TPM normalization, and all arrays were subjected to RMA normalized in GSE62452 and GSE28735. A total of 104 circadian clock-associated genes that were retrieved in MSigDB database [19]. Basic information for gene expression data was shown in the Table 1.

**Table 1:** Basic information for gene expression data.

**Database**	**Normalization method**	**Samples with OS**	**Survival time type**
Training set	TPM	120	OS
Validation set	TPM	52	OS
GSE62452	RMA	65	OS
GSE28735	RMA	42	OS

TPM, transcripts per million; RMA, robust multi-array analysis; OS, overall survival.

### Establishment and evaluation of the circadian clock-related indicator

A total of 104 circadian clock-related genes were fed into univariate Cox proportional hazards (PH) regression model, and then genes of *P*<0.05 were fed into LASSO regression to obtain the final fitted genes. The hazard of each individual was quantified using the final fitted gene expression and their own weight.

The performance of the model was quantified and compared based on the receiver operating characteristic (ROC) curve and Kaplan-Meier curve using the final fitted gene expression and their own weight.

### Differential expression analysis

To obtain the possible central genes, we executed differential expression analysis with the package edgeR in R. Raw counts were initially utilized to filter out those tremendously low-expressed genes, and then subjected to TMM normalization. Afterwards, processed data were characterized using negative binomial distribution and resulting differential expression calculation. Thresholds used here were *P* < 0.05 and |logFC| > 1. *P* < 0.05 is generally used as the significant relevance in the statistics, and |logFC| > 1 is regarded as an apparent fold change between the tumor and control groups in the bioinformatics.

### Characterization of intro-tumor immune cell types

Intra-tumor cell types were quantified by six different computational algorithms XCELL, TIMER, QUANTISEQ, MCPCOUNTER, EPIC, and CIBERSORT [20, 21]. The robustness of these approaches has been checked by laboratory assays. These estimation algorithms are basically based on two concepts, i.e., deconvolution and non-overlapping sets of gene lists which can represent for different immune infiltrates. Parameters used here were all in default Data used were TPM values.

### Quantitative analysis of circadian clock

Quantification of circadian clock was analyzed via single-sample gene set enrichment analysis [22]. Single-sample gene set enrichment analysis represents a computational strategy that can quantify a specific gene set for individual samples. Here, we used this mathematic strategy to estimate the levels of circadian clock for the comparison between the cancer group and the control group.

### Interrogation of physiological and pathological processes involved in interested genes

Physiological and pathological processes that were associated with interested gene lists were interrogated with R package “clusterProfiler” [23]. Physiological and pathological processes were mainly retrieved from two canonical databases: the gene ontology and Kyoto Encyclopedia of Genes and Genomes pathways.

### Statistics

Statistics were completed with R language. *t* test was used for continuous data. Pearson’s correlation analysis was utilized to analyze the association between two continuous variables. *P* value less than 0.05 was considered statistically relevant.

### Data availability

All data in this article are available from the public databases, and are freely available to any scientist wishing to use them for noncommercial purposes, without breaching participant confidentiality. Further information is available from the corresponding author on reasonable request.

## RESULTS

### Development of a circadian clock-related prognostic signature

**Figure 1:**
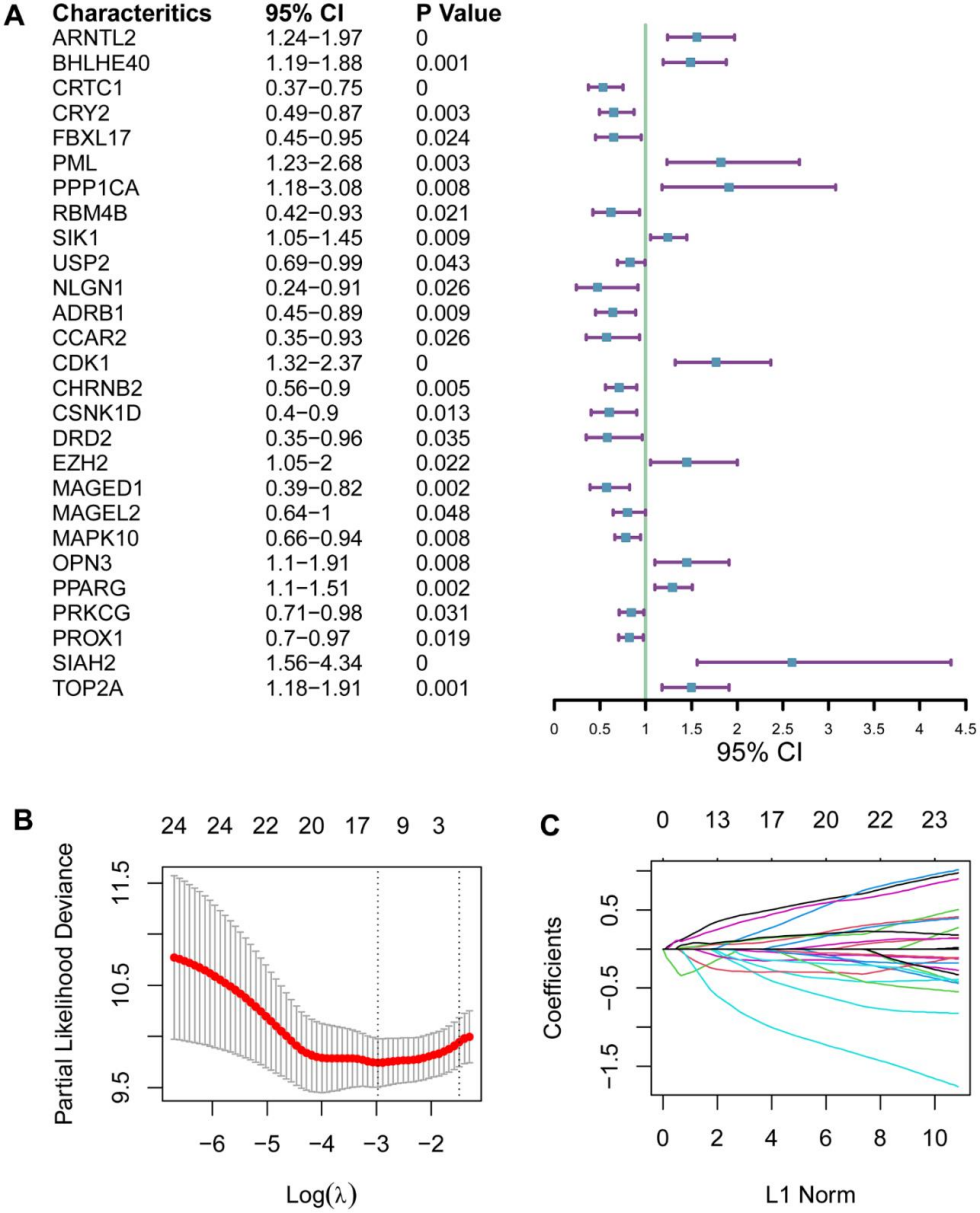
**Development of a circadian clock-related prognostic signature.** (**A**) Forest plot for 27 genes with a statistical relevance. (**B**, **C**) The 27 qualified genes were subsequently fed into LASSO regression model.

Circadian clock endows humans the ability to perceive temporal changes of the external environment through a series of physiological activities, and circadian rhythm disruption increases the hazard of a series of conditions such as metabolic disorders, cardiovascular diseases and cancer. To better distinguish between pancreatic ductal adenocarcinoma (PADA) patients with good prognosis and poor prognosis, we intended to develop a circadian clock-related indicator of prognostic outcomes. We analyzed 104 circadian clock-related genes in univariate Cox proportional hazards (PH) regression model, and 27 genes displayed a statistical relevance (*P*<0.05; Figure 1A). The 27 fitted genes were subsequently fed into LASSO regression model. 13 genes (*ARNTL2, BHLHE40, FBXL17, FBXL8, PPP1CB, RBM4B, ADRB1, CCAR2, CDK1, CSNK1D, KLF10, PSPC1, SIAH2*) were finally selected for the development of a prognostic signature (Figure 1B, 1C), which could be exploited to the quantification of the prognostic hazard of PADA patients via an equation based on the mRNA levels and the weights of the selected genes: *ARNTL2*×0.444 + *BHLHE40*×0.07 + *FBXL17*×-0.249 + *FBXL8*×-0.143 + *PPP1CB*×0.157 + *RBM4B*×-0.167 + *ADRB1*×-0.022 + *CCAR2*×-0.289 + *CDK1*×0.108 + *CSNK1D*×-0.847 + *KLF10*×0.116 + *PSPC1*×-0.087 + *SIAH2*×0.362.

### Evaluation of the performance of the circadian clock-related signature

**Figure 2:**
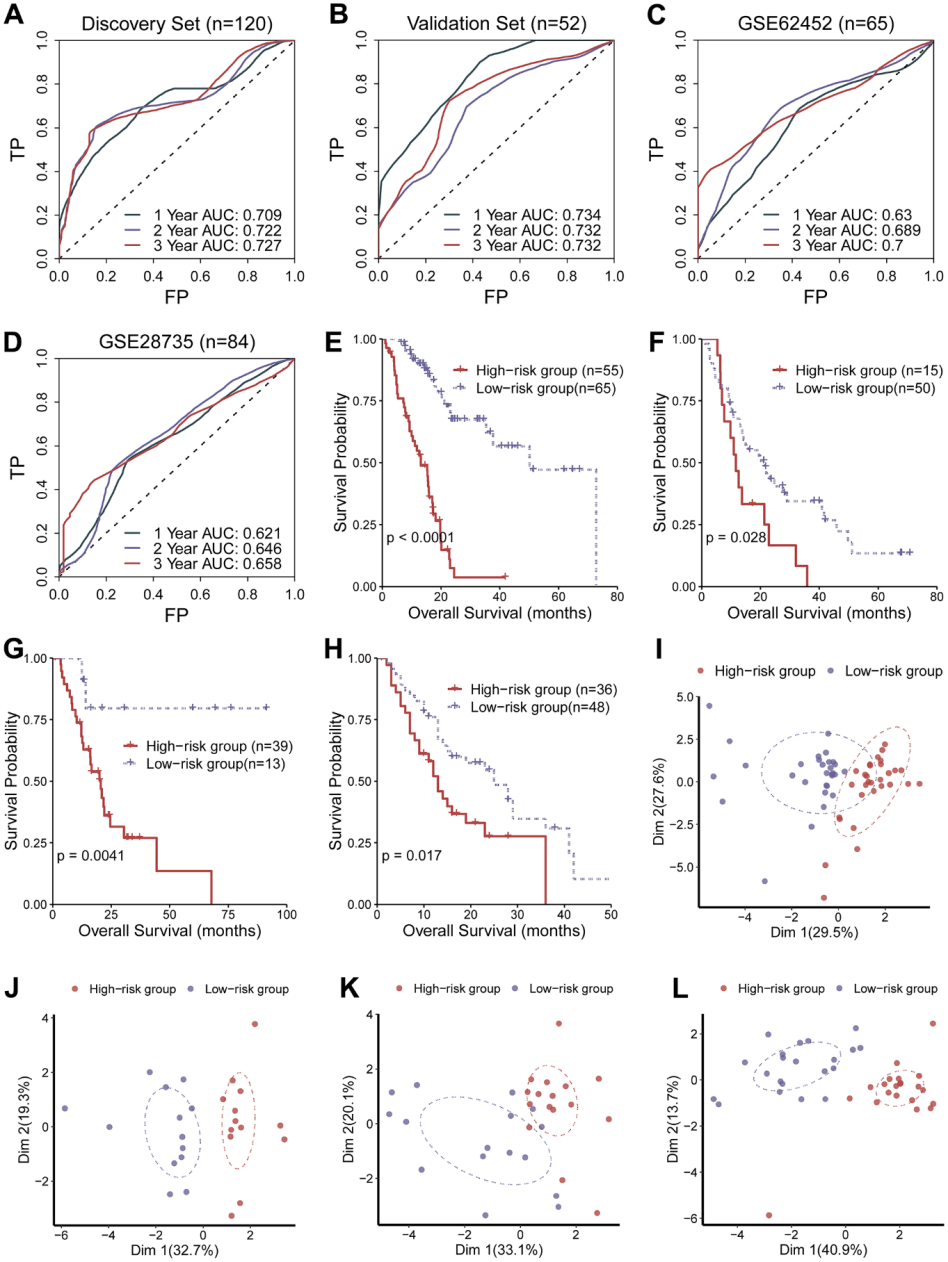
**Evaluation of the performance of the circadian clock-related signature.** (**A**–**D**) Area under the curve (AUC) for predicting 5-year overall survival were 0.727, 0.732, 0.700, and 0.658 in the training set, the validation set, and the two test sets, respectively. (**E**–**H**) KM analysis revealed that the low-risk population had desirable prognostic outcomes compared with the high-risk population in the training set, the validation set and the two external test sets. (**I**–**L**) PCA analysis demonstrated that the low-hazard cohort were clearly separated from the high-hazard cohort in Dimension 1.

Performance of the circadian clock-related signature was checked in the discovery set (n = 210), validation set (n = 52), GSE62452 (n = 65), and GSE28735 (n = 84), using three distinct statistical approaches: receiver operating characteristic (ROC) curve, Kaplan-Meier (KM) curve and principle component analysis (PCA). Area under the curve (AUC) for predicting 5-year overall survival were 0.727, 0.732, 0.700, and 0.658 in the training set, the validation set, and the two test sets, respectively (Figure 2A–2D), suggesting its predictive ability. KM analysis revealed that the low-risk population had desirable prognostic outcomes compared with the high-risk population in the training set, the validation set and the two external test sets (log-rank test, *P* <0.05; Figure 2E–2H). Concordantly, PCA analysis demonstrated that the low-hazard cohort were clearly separated from the high-hazard cohort in Dimension 1 (Figure 2I–2L). Altogether, these results supported that the circadian clock-related gene signature possessed a prognostic ability. To find out whether there was a significant variation in disease severity between the discovery cohort, validation cohort and test cohort, we compared histologic stage and tumor stage between these datasets. The result showed there was no significant different between the discovery cohort and the validation cohort, while the test set had a more severity of histologic stage and tumor stage (Supplementary Figure 1A, 1B). Moreover, we calculated the C-index of the clock-related signature in each cohort. C-index was 0.754, 0.747, 0.682 and 0.701 in the discovery cohort, validation cohort, GSE62452, and GSE28735, respectively. These results demonstrate that the parameters of this model were consistent across different populations, further emphasizing the stability and usefulness of the model. Furthermore, we analyzed the relationship of the established signature with clinical data (TNM staging), and found that there were no significantly difference across TNM staging (Supplementary Figure 1C–1E), implying that current TNM staging might not be an ideal indicator for predicting clinical endpoints.

### Signaling transduction processes associated with the predictive signature

**Figure 3:**
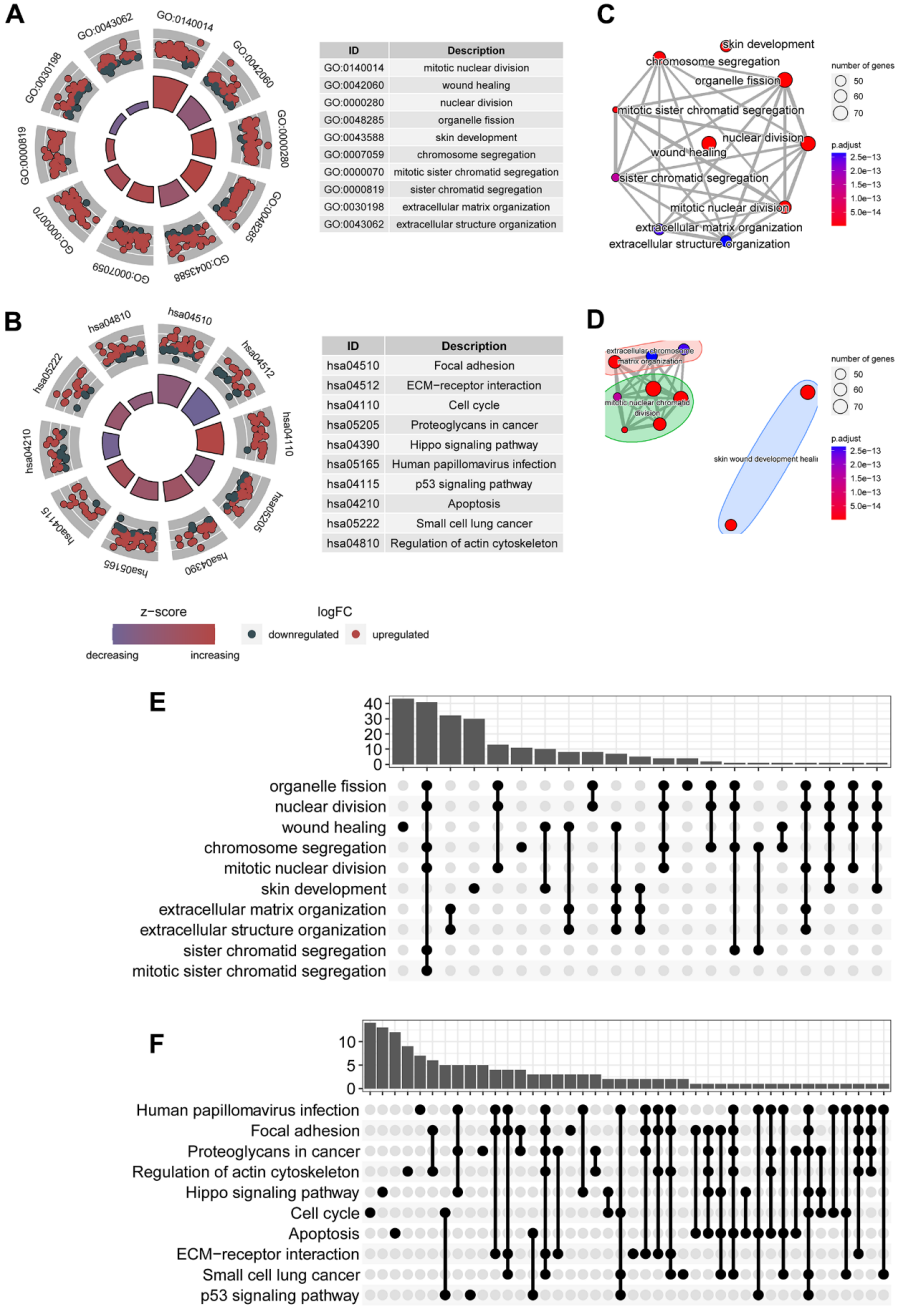
**Signaling transduction processes associated with the predictive signature.** (**A**) Top ten gene ontology terms associated with circadian clock were shown, including mitotic nuclear division, wound healing, chromosome segregation. (**B**) Top ten KEGG signaling pathways associated with circadian clock were shown, including focal adhesion, ECM-receptor interaction, cell cycle, p53 signaling pathway, and apoptosis. (**C**) Crosstalk communication between these physiological and pathological processes showed these pathways are mutually related. (**D**) Multiple common genes were observed in these biological processes and signaling pathways. (**E**) There existed multiple shared genes in top 10 biological processes, implying a crosstalk between these biological functions. (**F**) There existed multiple shared genes in top 10 KEGG signaling pathways, suggesting an interaction between these pathways.

To explore physiological and pathological processes that were associated with the predictive signature, we executed functional enrichment analysis for genes that were mostly associated with the predictive signature. Top ten gene ontology terms were mainly mitotic nuclear division, would healing, nuclear division, and organelle fission (Figure 3A). Top ten signaling transduction processes were mainly focal adhesion, ECM-receptor interaction, cell cycle, proteoglycans in cancer, hippo signaling pathway, p53 signaling pathway, and apoptosis (Figure 3B). Subsequently, crosstalk communication between these physiological and pathological processes were interrogated, and the results showed that chromosome segregation was associated with organelle fission, mitotic sister chromatid segregation and extracellular structure organization (Figure 3C). Besides, these physiological and pathological processes can be clustered into three clusters (Figure 3D). Multiple common genes were observed in these biological processes and signaling pathways (Figure 3E, 3F), furtherly implying crosstalk networks among these pathways.

### Association of the circadian clock-related signature with intra-tumor immune infiltrates

**Figure 4:**
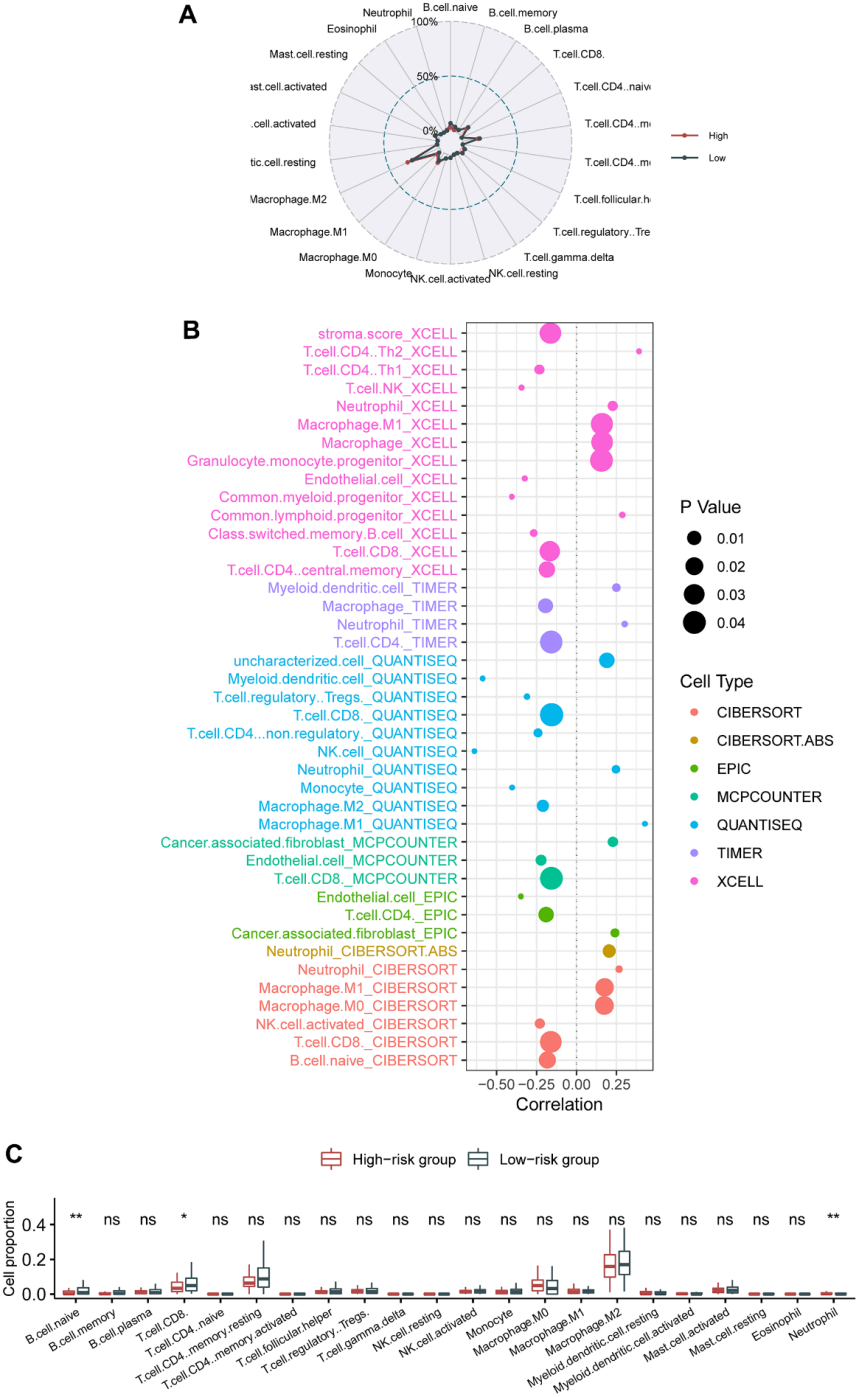
**Association of the circadian clock-related gene signature with intra-tumor immune infiltrates.** (**A**) Radar plot showed no apparent switch between the low- and the high-risk cohorts. (**B**) Bubble chart revealed that CD8 T cell and B cell were critically negatively correlated with the hazard. (**C**) Box plot demonstrated that CD8 T cell, B cell, and neutrophils were significantly switched between the high-risk patients than in the low-risk patients.

We interrogated the shift in the fractions of various immune cell types between the low- and the high-risk cohorts. Radar plot showed no apparent switch between the low- and the high-risk cohorts (Figure 4A). Nevertheless, bubble chart revealed that CD8 T cell and B cell were critically negatively correlated with the hazard (Figure 4B). Likewise, box plot demonstrated that CD8 T cell and B cell were critically reduced in the high-risk cases compared with in the low-risk cases, whereas neutrophils were significantly augmented in the high-risk patients than in the low-risk patients (Figure 4C).

### Association of the circadian clock-related signature with intra-tumor immune signaling pathways

**Figure 5:**
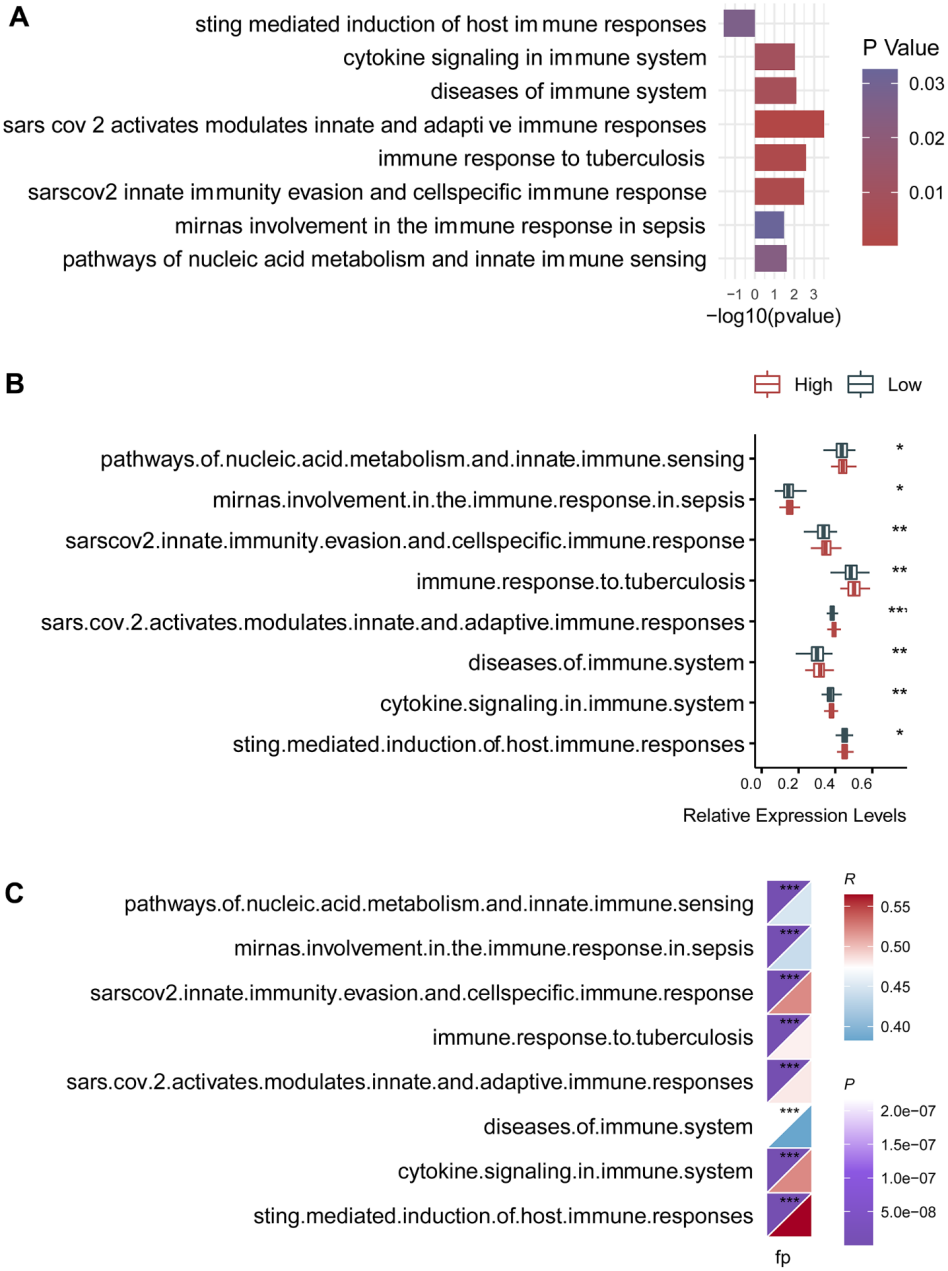
**Association of the circadian clock-related gene signature with intra-tumor immune signaling pathways.** (**A**) There were eight differentially expressed signaling pathways. (**B**) Box plot demonstrated a consistent change in the expression levels of these immune pathways. (**C**) The risk score was also significantly correlated with these eight immune pathways.

To further investigate the changes of immune function between the high- and the low-risk groups, we selected 25 immune-related signaling pathways from 3049 canonical pathways, and calculated their expression levels for individual patients both in the high- and the low-risk groups. Then, we found that there were eight differentially expressed signaling pathways (Figure 5A). Among them, there were seven immune-related signaling pathways that were upregulated in the high-risk group, including cytokine signaling in immune system, diseases of immune system, immune response to tuberculosis, and pathways of nucleic acid metabolism and innate immune sensing. Box plot demonstrated a consistent change in the expression levels of these immune pathways (Figure 5B). Remarkably, the risk score was also significantly correlated with these eight immune pathways, further highlighting the effect of the circadian clock-related gene signature on the immune system (Figure 5C).

### Association of the gene signature with response to molecular targeted therapy in pancreatic ductal adenocarcinoma

**Figure 6:**
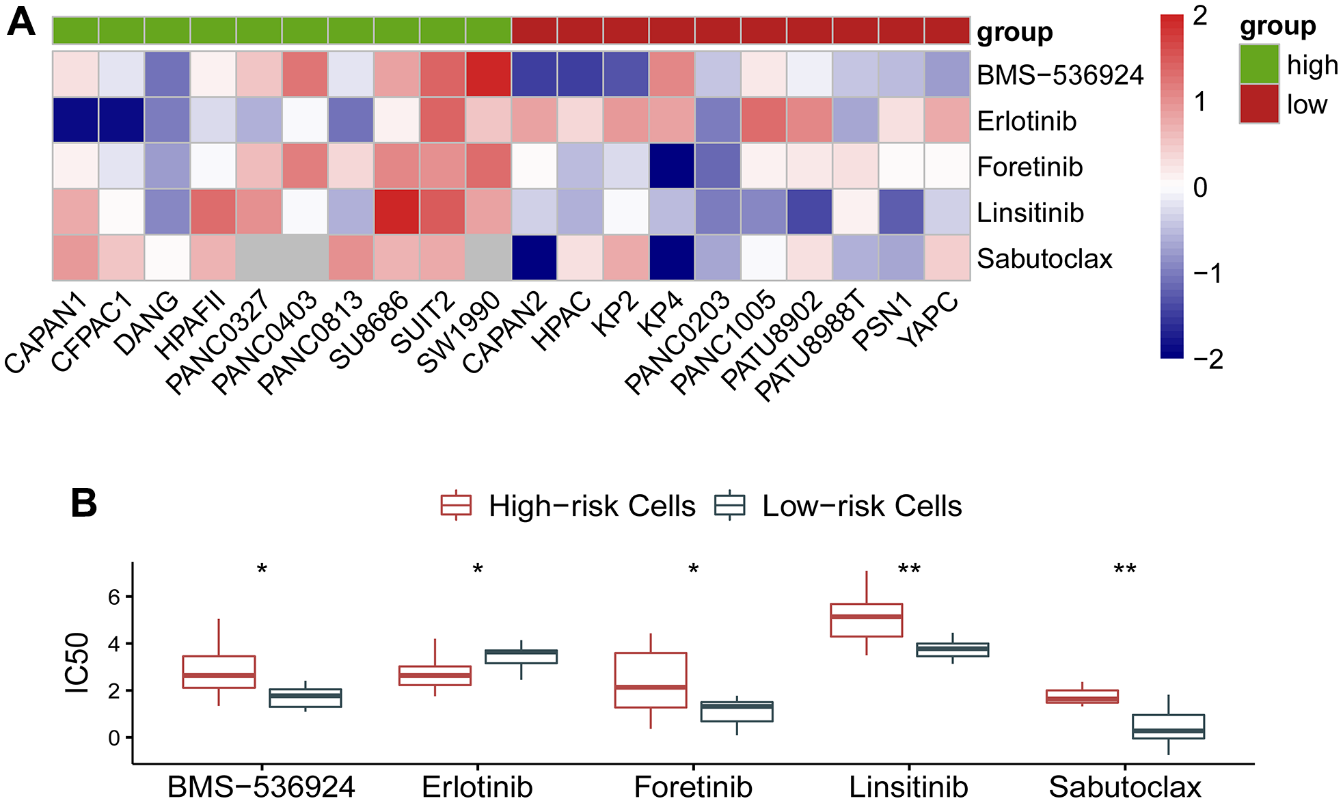
**Association of the gene signature with response to molecular targeted therapy in pancreatic ductal adenocarcinoma.** (**A**) IC50 of BMS-536924, Erlotinib, Foretinib, Linsitinib, and Sabutoclax exhibited a distinct effect on the low- and the high-risk cohorts. (**B**) Quantitative analysis showed that BMS-536924, Foretinib, Linsitinib, and Sabutoclax were more sensitive in the low-risk cancer cells, whereas Erlotinib was more effective in the high-risk cancer cells.

As the circadian clock-related prognostic signature showed its performance in predicting survival and exhibited an effect on immune response, we naturally proposed a hypothesis whether the gene signature could reflect the response to molecular targeted therapy in cancer. To clarify this problem, we investigated drug response of pancreatic cancer cells to a variety of agents using data from GDSC and CCLE database. As expected, the circadian clock-related signature manifested an association with drug response. IC50 of BMS-536924, Erlotinib, Foretinib, Linsitinib, and Sabutoclax exhibited a distinct effect on the low- and the high-risk cohorts (Figure 6A). Quantitative analysis showed that BMS-536924, Foretinib, Linsitinib, and Sabutoclax were more sensitive in the low-risk cancer cells, whereas Erlotinib was more effective in the high-risk cancer cells (Figure 6B). The specific information on these agents was described in Table 2.

**Table 2:** Basic information on agents.

**Drug**	**Targeted molecules**	**Targeted pathways**
BMS-536924	IGF1R, IR	IGF1R signaling
Erlotinib	EGFR	EGFR signaling
Foretinib	MET, KDR, TIE2, VEGFR3/FLT4, PDGFR, EGFR	RTK signaling
Linsitinib	IGF1R	IGF1R signaling
Sabutoclax	BCL2, BCL-XL, BFL1, MCL1	Apoptosis regulation

## DISCUSSION

We developed a circadian clock-related prognostic signature that could distinguish pancreatic ductal adenocarcinoma (PADA) patients with unwanted prognostic outcomes from those with desirable prognostic outcomes and be reflective of response to molecular targeted therapy. Remarkably, the ability of the current signature to predict survival outcomes was independently verified in two external data sets, stratifying PADA patients into the low- and the high-risk groups, outperforming current pathological staging system, implying that this circadian clock-related gene signature could serve as a prognostic tool for PADA patients.

Pancreatic cancer represents a fatal condition with a poor prognosis. Surgical removal is still the standard treatment for patients with a resectable lesion [24]. Nevertheless, the option for operation must be chosen cautiously due to a relatively low postoperative mortality and a relatively high incidence of postoperative complications [25–27]. Therefore, it is necessary to identify PADA patients who are suitable for surgery or not. Here, we established this circadian clock-associated signature could be exploited as such an indicator for risk prediction, which would hopefully aid in the formulation of treatment strategies.

Of the 13 genes identified to constitute the prognostic gene signature, part of them do not have an obvious function in cancer. *ARNTL2*, *BHLHE40* are reported to promote pancreatic ductal adenocarcinoma progression [28, 29]. Interestingly, *BHLHE40* can drive pro-tumor neutrophils with hyperactivated glycolysis in pancreatic ductal adenocarcinoma. *FBXL17*, *FBXL8*, *CDK1*, *CSNK1D*, and *PSPC1* have not been well studied in pancreatic ductal cancer. However, several of them have been implicated in pancreatic cancer, with a relatively high expression and a poor survival [30–33]. These suggest that the 13 genes in this prognostic signature are worth further investigation for their potential as novel therapeutic targets.

As above mentioned, neutrophils are associated with the proliferation and metastasis of cancer in pancreatic cancer [34, 35]. Consistently, we identified that neutrophils were also elevated in the high-risk group than in the low-risk group. Besides, neutrophil extracellular traps could mediate resistance to checkpoint blockade in pancreatic cancer [36], and targeting neutrophils could prevent pancreatic cancer metastasis [37] and improve effects of immune checkpoint blockade [38].

Besides, our analyses demonstrated that BMS-536924, Foretinib, Linsitinib, and Sabutoclax were more sensitive in the low-risk group, whereas Erlotinib was more effective in the high-risk group. BMS-536924 is reported to has anti-neoplastic activity and can sensitize cancer cells to molecular target drugs [39, 40]. However, its role in pancreatic cancer is required to be investigated. Likewise, Foretinib showed an anti-tumor function [41], while its relationship with drug sensitivity remains elusive. Combination therapy based on Sabutoclax and minocycline was more effective *in vivo* and *vitro* experiments [42].

We have to admitted that a prominent limitation in the present research is a lack of *in vivo* and *in vitro* assays to further support the findings of this study or interrogate the mechanisms underlying the roles of the signature and pivotal genes in pancreatic cancer. Further laboratory experiments are required to solve this limitation.

Collectively, we built a novel circadian clock-related signature, which can stratify patients of distinct prognostic outcomes and be reflective of therapeutic effects of molecular targeted therapy. These findings could incorporate the pharmacological modulation of circadian clock into future therapeutic strategies.

## Supplementary Material

Supplementary Figure 1
