# Supplementary material for: Profiling of a novel circadian clock-related prognostic signature and its role in immune function and response to molecular targeted therapy in pancreatic cancer
Source: Aging (Albany NY). 2023 Jan 9;15(1):119–33. doi: 10.18632/aging.204462 (PMC9876629; doi:10.18632/aging.204462)
Supplement: Supplementary Figure 1 [file aging-15-204462-s001.pdf]

SUPPLEMENTARY MATERIALS

Supplementary Figure

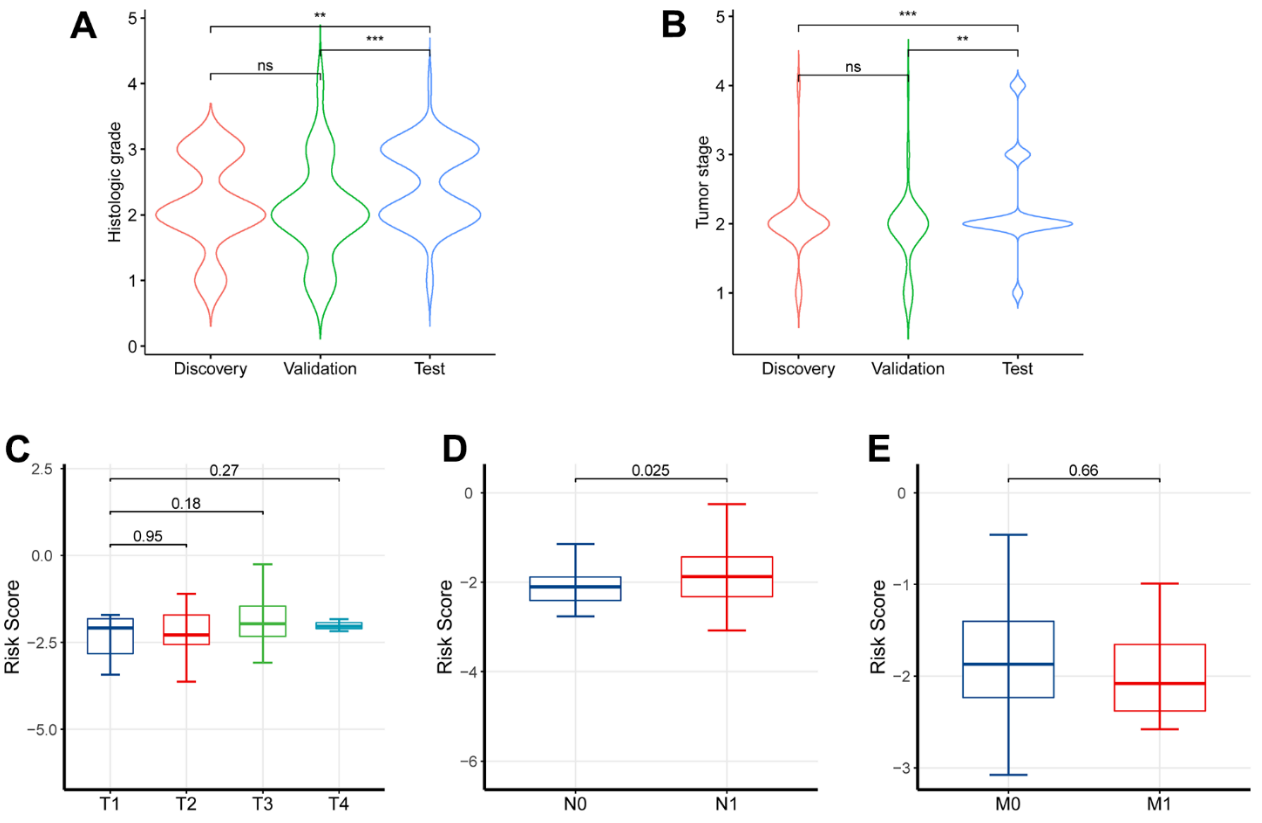

Supplementary Figure 1. The association of current staging system with the signature. (**A**) Test set showed a more severity of histologic stage compared with discovery and validation set. (**B**) Test set showed a more severity of clinical grades compared with discovery and validation set. (**C**–**E**) There were no significantly difference of risk scores between different TNM stages, implying that current TNM staging might not be an ideal indicator of predicting clinical endpoints.
